# A New method for simultaneous qualitative and quantitative determination of amlodipine besylate and atorvastatin calcium in bulk and pharmaceutical formulations using transmission FT-IR spectroscopy

**DOI:** 10.1016/j.heliyon.2023.e14189

**Published:** 2023-03-03

**Authors:** Sherwan Ibesh, Yaser Bitar, Saleh Trefi

**Affiliations:** Department of Pharmaceutical Chemistry and Quality Control, Faculty of Pharmacy, University of Aleppo, Aleppo, Syria

**Keywords:** Amlodipine besylate, Atorvastatin calcium, Caduet®, FTIR

## Abstract

A simple, precise, rapid, and eco-friendly FTIR spectrophotometric method was developed and validated for simultaneous analysis of amlodipine (AML) and atorvastatin (ATV) in drug combination preparations. Firstly, synthetic mixtures were made and scanned with FTIR instrument. Then the result spectra were converted automatically to absorbance spectra. The calibration model was made depending on Beer's law which relates concentration to absorbance. Two characteristic bands corresponding to the carbonyl group at 1708-1688 cm^−1^ and 1660-1632 cm^−1^ for AML and ATV, respectively, were selected for quantification. The absorbance of a series of standards was measured as the AUC of the chosen bands. Then, the calibration line was obtained by plotting the measured AUCs and the actual concentrations against each other. Validation tests were performed per ICH recommendations. Specificity was evaluated by the separation of APIs and excipients from marketed preparations by methanol. Then, the spectra of extracted excipients, APIs, and pharmaceutical samples were taken and overlapped. The selected peaks were specific and did not interfere with each other or other peaks from the excipients used in the tablet's matrix. Linearity for AML and ATV was in the range of 0.1–1% w/w with excellent coefficients of determination (R^2^), 0.998 and 0.999 for AML and ATV, respectively. The proposed analytical method was accurate and precious, as the RSD values were less than 2%. The proposed FTIR method was successfully applied to estimate the exact quantity of APIs in pharmaceutical samples. Recoveries were accepted in accordance with USP and were in the range of 94.62–100.6% and 98.175–101.06% for AML and ATV, respectively. Likewise, the acquired results were compared with the HPLC method. And the t- and F- tests were calculated and compared with the theoretical values, which indicate the similarity of results in both developed and reported methods.

## 1 Introduction

**Fig. 1:**
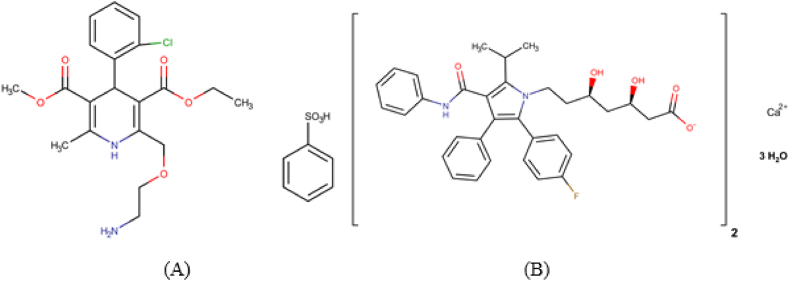
Chemical structure of A) AML besylate and B) ATV calcium hydrate.

Amlodipine Besylate (AMP), chemically: 3-ethyl 5-methyl (4RS)-2-[(2-aminoethoxy) methyl]-4-(2-chlorophenyl)-6-methyl-1,4-dihydropyridine-3,5 dicarboxylate benzene sulfonate (empirical formula C_20_H_25_ClN_2_O_5_.C_6_H_6_SO_3_) [1] Fig. 1a, is an oral dihydropyridine, which affects the vascular calcium channels much greater than cardiac calcium channels, and consequently it is, predominantly, useful in the management of hypertension. It is also useful in managing variant angina because of its vasodilator effect. The dihydropyridines exert their action, preventing calcium from entering the cells by binding to L-type calcium channels in the heart and the smooth muscle of the coronary and peripheral arteriolar vasculature [2]. Amlodipine is marketed as the benzene sulfonic acid salt (besylate) under the name Norvasc® [3] Atorvastatin Calcium (ATV), chemically: (3.R, 5R)-7-[2-(4-fluorophenyl)-5-(1-methyl ethyl)-3-phenyl-4-(phenyl carbamoyl)-1H-pyrrol-1-yl]-3, 5-dihydroxy heptanoate trihydrate (empirical formula C_20_H_25_ClN_2_O_5_.C_6_H_6_SO_3_.3H_2_O) [1], Fig. 1b. The main effect of atorvastatin is lowering LDL through a mevalonic acid-like moiety that competitively inhibits HMG-CoA reductase. Therefore, inhibits a first and key rate-limiting step in cholesterol biosynthesis in the liver. Atorvastatin is used for the management of high cholesterol [4]. Fixed-dose drug combinations are used to increase patient compliance and reduce costs. The combination of atorvastatin and amlodipine was first introduced into the market by Pfizer under the name of Caduet® in 2004 [5]. Caduet® was approved in the U.S. for treating hypertension and coronary artery disease (CAD) and as an adjunct therapy to diet for the prevention of cardiovascular disease and hyperlipidemias [6]. AML and ATV are both official in BP and USP [1,7]. The revision bulletin of the USP-NF describes an HPLC method for the simultaneous determination of both AML and ATV [8].

A detailed literature survey revealed many analytical methods for the simultaneous estimation of amlodipine besylate and atorvastatin calcium in pharmaceutical dosage forms. These methods include chromatography [[9], [10], [11], [12], [13], [14], [15], [16], [17], [18], [19]], capillary electrophoresis [20], and spectrophotometric methods [[21], [22], [23], [24], [25], [26], [27]]. All these techniques usually require high-cost analysis as they require large volumes of solvents and long analysis time due to lengthy and difficult sample preparation like the mobile phase preparation, which makes them unsuitable for rapid analysis of bulk samples on an industrial scale. On the other hand, FT-IR spectrometers have been growing in popularity as they offer speed, accuracy, and high sensitivity, which was impossible to achieve with other spectrometers. This technique allows rapid analysis of micro-samples precise to the nanogram level in some instances, making the FT-IR an essential tool for problem-solving in many studies [28].

Previously, FT-IR spectroscopy was regarded as a qualitative technique. However, remarkable quantitative work was published recently [[29], [30], [31], [32]].

To the best of our knowledge, the FTIR method has not been reported for simultaneous determination of both AML and ATV. Therefore, the endeavor was made to develop a simple, cost-saving, eco-friendly, rapid, and non-destructive method using FTIR to quantify AML and ATV content in bulk and tablet formulations. The method was validated in accordance with the International Conference on Harmonization ICH guideline [[33], [34], [35]].

## 2 Experimental

### 2.1 Chemicals and reagents

Standard analytical grade samples of amlodipine besylate with a purity of 99.88% were obtained as a gift from Lee pharma ltd, India.
Standard analytical grade samples of Atorvastatin calcium with a purity of 99.14% were obtained as a gift from Reine Life sciences Gujarat, India.

For recording spectra, the IR spectroscopic grade of potassium bromide (99,999%) was obtained from Merck KGaA, Germany. KCl was previously dried each time before using in the oven at 120° for 2 h.

Different Amlostine® tablets (Manufactured by Al Nawras Pharma, Hama, Syria), stated to contain different ratios of AML and ATV per tablet (5 or 10 mg for AML and 10, 20, 40, or 80 mg for ATV), were purchased from local community pharmacies (Aleppo, Syria).

HPLC-grade methanol was obtained from Scharlau, Spain.

HPLC-grade acetonitrile was obtained from Biosolve, France.

HPLC-grade phosphoric acid was obtained from Merck, Germany.

Double distilled water.

### 2.2 Equipment and software

The spectrum of standards and samples in the tablet form was obtained by Alpha Bruker FTIR spectrometer equipped with KBr optics and deuterated triglycine sulfate (DTGS) detector. The instrument was controlled by instrument-specific IR spectra analysis software package OPUS 6.5 for data processing. Infrared spectra of samples were recorded in the range of 4000–400 cm^−1^. The resulting spectrum was the average of 16 scans at a resolution of 4 cm^−1^. Background measurement was done using a freshly prepared KCl disc before each single sample measurement.

Chromatographic analysis was done with an HPLC system consisting (of Agilent 1260 infinity, Germany) equipped with a UV detector, auto-injector, and vacuum degasser. The chromatographic conditions were:

The detection wavelength is 246 nm.

The column: C-18 (15 cm × 4.6 mm, 5 μm).

The flow rate is 1.0 ml/min.

The injection volume is 20 μl.

### 2.3 Calibration curve construction

A stock mixture powder of equal concentration of AML and ATV in KCl was prepared. Then, the stock mixture was used to separately prepare seven AML and ATV mixture standards by diluting a particular quantity from the primary stock mixture powder by KCl to the desired weight. The prepared standard concentrations ranged between 0.10% and 1.00% w/w in KCl pellet, and 100 mg of the mixture was condensed into 13 mm die at a pressure of 10 tons for 2 min. After that, each standard was measured on FTIR and repeated three times. Next, the mean spectrum was taken and converted to absorbance (AB). Finally, one band for every component was selected, and area under curve (AUC) values were measured and plotted versus standard concentrations to obtain a calibration curve.

Using OPUS Analyst software 6.5, a calibration curve based on standard spectra was built. A Specific region of 1708-1688 cm^−1^ for AML and 1660-1632 cm^−1^ for ATV was selected for the best results.

### 2.4 Sample preparation procedure

Twenty tablets were each weighed accurately, and the average weight was calculated. All tablets were then grounded until homogeneous, and a quantity equivalent to 0.50 mg ATV was sampled. Next, an appropriate amount of the AML standard was added to the sample to make an equal concentration with atorvastatin. Then, the sample was diluted to 100 mg by potassium chloride to get 0.50% w/w concentration for both AML and ATV and condensed into 13 mm die at 10 tons for 2 min. Then, the resulting disc was scanned on an FTIR instrument to record spectra. AML and ATV content in the sample was measured using the calibration curve. The results of determining the contents were compared to the contents stated on the packaging.

### 2.5 Validation of analysis method

The developed method was validated concerning the following parameters: specificity, linearity, accuracy, precision, range, LOD, and LOQ. The parameter tests were done in accordance with the instructions mentioned in these references [[33], [34], [35]].

#### 2.5.1 Specificity

The spectrum of the active substances, the matrices, and the pharmaceutical sample were overlaid with each other. A range of particular wave numbers for each substance, where the peak present gives the best linear response and minimum interference, was selected and measured.

#### 2.5.2 Linearity and range

The linearity was evaluated by analyzing a set of standards of an equal concentration of AML and ATV. The concentration of the calibration set ranged between 0.1 and 1.0% w/w, so it covered the range of 20–200% of the test concentration (0.5%). Each concentration was measured three times, and the average was taken. Then, the AUC value of the selected peak was measured. A curve between standard concentration and AUC value was plotted, and the correlation coefficient (r) was calculated.

#### 2.5.3 Accuracy

In this test, the standard addition method was used. The results were evaluated after samples measuring and calculating the percent recovery from added analytes. Firstly, one tablet having an equal amount of AML and ATV was crushed into powder. Then, an appropriate quantity was sampled and measured to be used as a blank. After that, a standard mixture was added (1:1). The final blend was diluted with KCl to prepare three mixtures with active substance content of 80, 100, and 120% of the target concentration. Six individually prepared replicates at each level were measured. After that, the mean recovery, SD, and RSD were calculated. The method is accurate if recovery is in the range of 98–102% from added standard.

#### 2.5.4 Precision

The precision of the proposed method was evaluated by two parameters: repeatability (intra-day) and intermediate precision (inter-day). Repeatability test was done by measuring 100% of the test concentration using FTIR ten times on the experimental conditions. However, an intermediate precision test was done by measuring three concentration levels (80%, 100%, and 120%) from the test concentration repeated on two days. The AUC value of the selected peak was measured, then the measured value content of each standard was determined using the calibration curve. The analysis results obtained on two days should have a statistical RSD ≤2% as the acceptance criterion.

#### 2.5.5 Limit of detection and quantification

The selected peaks' area for quantification was measured for decreasing low-concentration standard mixtures until the analyte-related band vanished entirely. The lowest concentration which made a considerable band was investigated and analyzed eleven times. Then, LOD and LOQ value was calculated using equations (1), (2)), respectively:

(1) LOD=3×SD×C/M

(2) LOQ=10×SD×C/M

SD = the standard deviation C = the concentration of the standard mixture M = the mean band area.

### 2.6 Procedure for the determination of the pure powder by HPLC method

Different volumes from the working standard were pipetted into 50 ml standard volumetric flasks and diluted up to the mark with mobile phase to achieve final linearity concentrations. The preparation was injected into the chromatograph. The mobile phase, which consisted of (phosphate buffer: acetonitrile: methanol in the ratio of 53:43:4 v/v) was passed first for baseline correction with a flow rate of 1 ml/min and UV detection at 246 nm. After that, the peak response for each drug and concentration was recorded and used to construct the calibration curves by plotting the peak area versus the concentrations of each drug. Then, the linear regression equation of each curve was used to quantify the amount of the drug.

### 2.7 Procedure for analysis of marketed formulations by HPLC method

Four marketed formulations containing different ratios of AML and ATV were analyzed by the HPLC method. For this purpose, twenty tablets of each formulation were crushed to obtain a powder. An appropriate amount of powder was carefully weighed and added to a 100 volumetric flask and solved by 50 ml of the mobile phase. Then sonicated for 30 min, and the volume was completed up to the mark with the mobile phase. Three equivalent volumes were pipetted into different vials. The final concentration of both AML and ATV was equal to the standard solution. The preparation was injected into a chromatograph and, peak responses were recorded. The regression equations were used to quantify AML and ATV in the marketed formulation.

## 3 Result and discussion

### 3.1 Development of APIs assay method

This work brings about some advantages of using FT-IR, like speed, ease, and efficiency for calculating the exact amount of active compounds during routine laboratory or quality control analysis of pharmaceuticals.

**Fig. 2:**
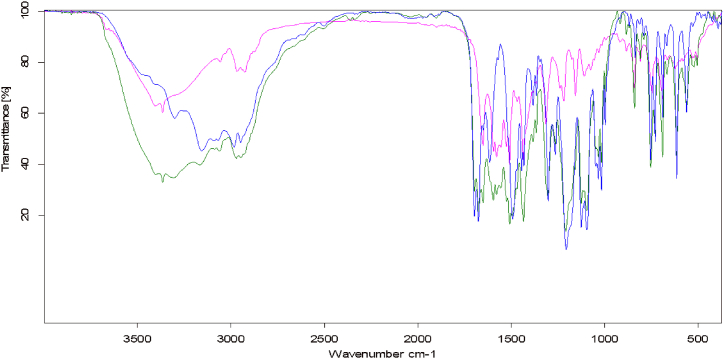
The transmission spectra of AML and ATV alone and a Mixture of 1:1 of them.

Depending on their structure, AML and ATV were expected to produce a typical spectrum in the infrared, such as the stretching of the –N-H (cyclic and aliphatic) and C=O, which does not often exist in the standard matrices of the tablets Fig. 2.

The spectra of a raw combination of AML and ATV were interpreted in Table 1, which shows the characteristic infrared absorption bands of each API with absorption location.

**Table 1:** Assignment for the characteristic infrared absorption bands of AML and ATV.

Functional Group assignment	Wave number (cm^−1^) for AML	Wave number (cm^−1^) for ATV	mixture
-OH (hydroxyl)	–	3364.70	3365.01
−NH	3299.13	3228.00	–
−C=O	1697.98–1674.92	1651.61	1697.40-1674.59-1651.01
−C-O	1017.62	–	1017.52
−CH (aromatic)	3068.28–3088.73	3056.09	3064.39
C=C (aromatic)	1615.72	1595.18	1615.27–1595.50

The band selected for quantitative analysis should give a strong peak, not affected with other peaks from other ingredients in the sample, be symmetrical and give a linear relationship versus calibration concentrations. C=O, N–H, or O–H groups are the most commonly used for quantitative purposes. The carbonyl stretching band is the most often used because it has high molar absorptivity and does not frequently overlap with other functional group bands. In addition, the carbonyl band is not as liable for chemical change or hydrogen bonding as hydroxide and amine groups [36].

**Fig. 3:**
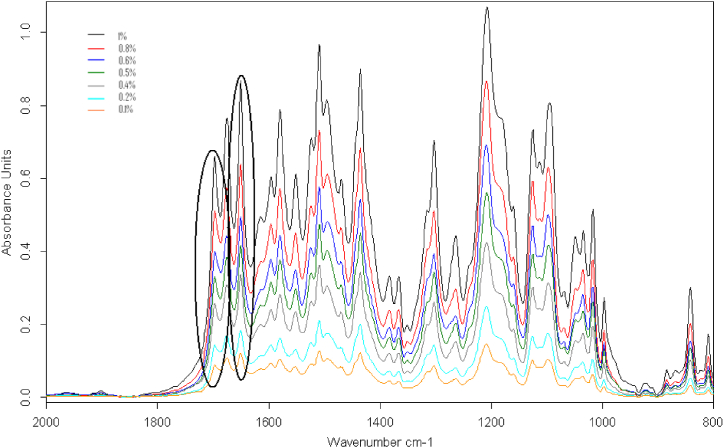
Set of FT-IR transmission spectra of the mixture standards of AML and ATV with selected bands for the quantitative analysis.

Two suitable peaks in the spectrum were chosen for calibration curve construction. One is centered by 1708-1688 with maximum absorption at 1697 for determination of AML, and the other is centered by 1660-1632 with maximum absorption at 1650 for determination of ATV. The peaks selected for analysis have high molar absorption because they correspond to the carbonyl group C–O of AML and amide carbonyl –NH–CO of ATV. Fig. 3.

**Fig. 4:**
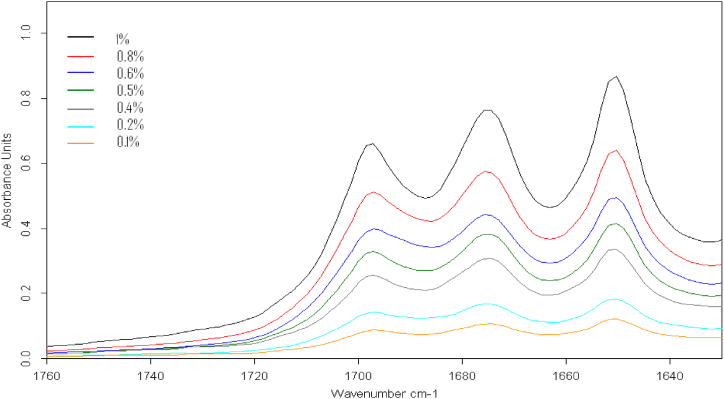
Overlay of carbonyl bands (1630-1760 cm-1) for mixture standards of various concentrations.

From the overlapped spectrum, shown in Fig. 4 below, it is concluded that peaks in the range of 1630–1700 cm-1 tend to be linear. These peaks correspond to the stretching of the C=O bond of AML and ATV.

**Fig. 5:**
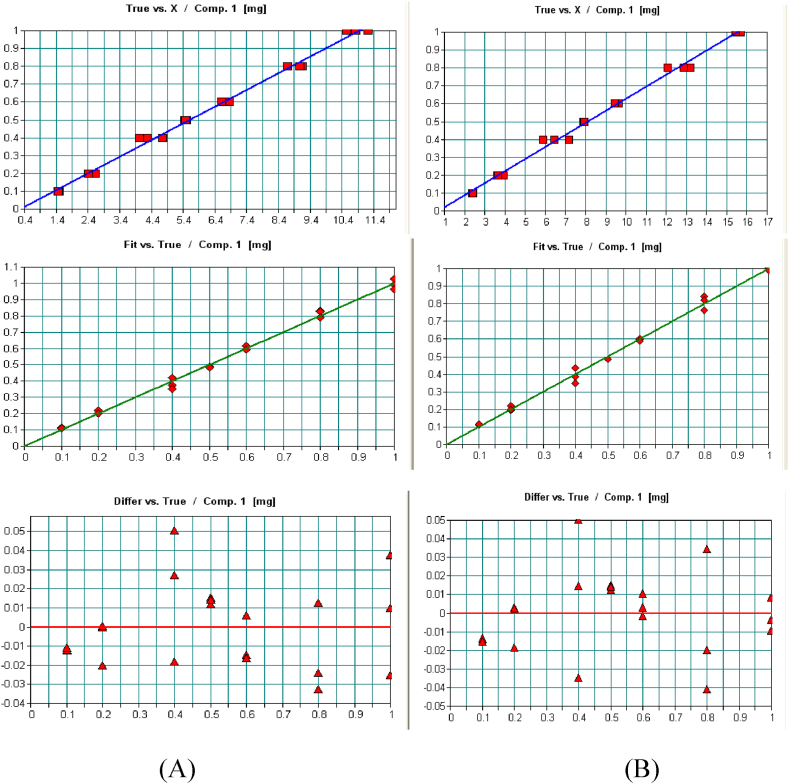
OPUS Analyst of AML and ATV standards in region 1708 - 1630 cm−1 of A) AML B) ATV.

In this work, firstly, the infrared light that passes the sample was recorded. Then the transmittance spectra of each standard were converted into absorption spectra and the baseline was automatically corrected by the instrument. Then, the data obtained was used by OPUS 6.5 Analyst to measure the band area mode A (the integration will be performed between the band, abscissa, and the frequency limits defined) since the manual calculation of a particular band area takes a long time and is a very arduous assignment. Therefore, the OPUS Analyst was used for spectra screening to generate the calibration easily and efficiently, saving a lot of time and labor. The quantitative analysis of a substance using FTIR is based on Beer-Lambert law, which states that the amount of absorbed light from the sample is directly proportional to the concentration of absorbing components in the sample. In this study, the absorbance is measured as carbonyl peak area, which was applied for the quantitative determination of AML and ATV in pharmaceutical formulations. The linear regression results showed an excellent linear correlation for the quantitative determination of both AML and ATV simultaneously as shown in Fig. 5A-B.

From the curves obtained above, the good accuracy of the FT-IR method can be concluded. That was because the % difference plot between the original and calculated values for the linearity range concentrations was in the range of 0.01–0.05 mg. In other words, the predicted concentrations of analytes in the calibrated model were close to the calculated concentrations and scattered within a little difference less than 0.05 mg from the actual concentration.

### 3.2 Method validation

#### 3.2.1 Specificity

**Fig. 6:**
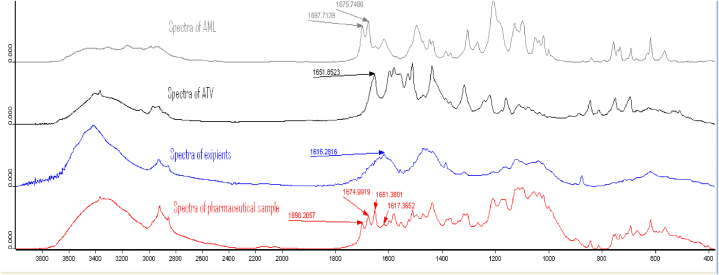
The results of Comparison of the spectra of APIs standard, excipients, and pharmaceutical sample.

The specificity test was carried out to determine whether there is any disturbance caused by the matrix components of the sample and to find out any interference between AML and ATV selected bands. The main excipients used in commercial tablets following the manufacturer were: calcium carbonate, microcrystalline cellulose, pregelatinized starch, croscarmellose sodium, and colloidal silicon dioxide. Excipients and API were separated from the marketed preparations of different brands mentioned above with methanol as most excipients do not dissolve in methanol except croscarmellose sodium which is used in tablets as a disintegrating agent and its percentage is relatively small. The undissolved powder which represents the mixture of excipients was scanned. Then, the spectra were converted to absorbance spectra. The results were next overlaid, including the standard spectra of APIs, excipients, and commercial tablets. Fig. 6.

From the comparison above, it was concluded that the region selected for quantification of AML contains no band in the ATV spectrum and the region selected for ATV quantification shows no band in the AML spectrum. Besides, the spectrum of the pharmaceutical sample contains both selected bands in the range which are well-separated prominent bands and not disturbed by the matrix. Consequently, it becomes so easy to quantify both analytes from a single spectrum.

#### 3.2.2 Linearity

Linearity was observed in the range of 1–10 mg.g-1 and tested for both APIs by standard measurements of three individually prepared replicates at each concentration with re-sampling in every measurement. After that, the average value of AUC was taken and then, plotted against the concentration. The measurementresults are shown in Table 2 and Table 3 with the regression curves supported shown in Fig. 4.

**Table 2:** Amlodipine besylate calibration curve data using AUC versus concentration.

Concentration (w/w %)	AUC(n = 3)^a^			Mean ± SD
	1	2	3	
0.10	1.47	1.48	1.48	1.48 ± 0.01
0.20	2.63	2.42	2.41	2.49 ± 0.10
0.40	4.74	4.26	4.01	4.34 ± 0.30
0.50	5.45	5.48	5.46	5.46 ± 0.01
0.60	6.84	6.85	6.62	6.77 ± 0.11
0.80	9.16	9.10	8.68	8.98 ± 0.21
1.00	10.54	11.21	10.85	10.87 ± 0.27
Equation for regression line: y = 10.608x + 0.3142				R = 0.9997

a Note AUC: area under the curve of absorbance.

**Table 3:** Atorvastatin calcium calibration curve data using AUC versus concentration.

Concentration (w/w %)	AUC(n = 3)^a^			Mean ± SD
	1	2	3	
0.10	2.42	2.40	2.40	2.41 ± 0.01
0.20	3.95	3.63	3.63	3.74 ± 0.15
0.40	7.16	6.43	5.90	6.50 ± 0.52
0.50	7.91	7.93	7.90	7.91 ± 0.01
0.60	9.64	9.57	9.46	9.56 ± 0.07
0.80	13.19	12.87	12.10	12.72 ± 0.46
1.00	15.43	15.60	15.71	15.58 ± 0.11
Equation for regression line: y = 14.775x + 0.7473				R = 0.9995

a Note AUC: area under the curve of absorbance.

A linear regression equation of AML and ATV was obtained from the data above. For AML, the regression equation was: y = 10.608x + 0.3142 with a correlation coefficient value (r^2^) equal to 0.998. Meanwhile, ATV has the linear regression equation: y = 14.775x + 0.7473 with a correlation coefficient value (r^2^) equal to 0.999.

Based on these data, it was concluded that the method has fulfilled the linearity acceptance criterion in the concentration range of 0.10%–10% w/w because the correlation coefficient (r^2^) for the standard concentration levels is more than 0.997.

#### 3.2.3 Accuracy

Accuracy is characteristically demonstrated as the nearness between the measured results and the expected theoretical value. The test was carried out in presence of a sample matrix. The test results were shown in Table 4.

**Table 4:** Accuracy data of AML and ATV.

Drug	Amount added	Average of AUCs* ± SD	Amount Found	Recovery (%)	Mean recovery ± SD	RSD (%)
AML	0.400	4.56 ± 0.12	0.400	100.00	100.03 ± 1.18	1.18
	0.500	5.54 ± 0.09	0.493	98.60		
	0.600	6.78 ± 0.16	0.609	101.50		
ATV	0.400	6.57 ± 0.15	0.394	98.50	99.46 ± 0.90	0.90
	0.500	8.07 ± 0.16	0.496	99.20		
	0.600	9.67 ± 0.14	0.604	100.67		

Note: AUC: area under the curve of absorbance spectrum, *All values are reported as mean (n = 6).

Based on the accuracy data above, recovery of three concentrations ranged between 98.60-101.50% and 98.50–100.67% for AML and ATV, respectively. Therefore, the recovery of the proposed method was in the acceptance range of 98–102%.

#### 3.2.4 Precision

The precision of an analytical method declared the closeness of the acquired results (degree of scatter) from multiple homogeneous samplings under specified conditions.

Two parameters were tested to assess the precision of the present method. They included repeatability and intermediate precision clarified below.

##### 3.2.4.1 Intermediate precision

Intermediate precision will show the repeatability of results within laboratories. The variability in results is caused by some factors like different days, different environmental conditions, different analysts, and different equipment. Intermediate precision was demonstrated on three days. Every day the sample was measured five times. Then, the mean, standard deviation, and RSD were calculated, with results shown in Table 5.

**Table 5:** Intermediate precision data of AML and ATV.

Drug		Conc.	Found concentration ± SD^a^	Recovery (%)	RSD (%)
AML	First day	0.400	0.395 ± 0.004	98.75	1.01
		0.500	0.502 ± 0.001	100.40	0.20
		0.600	0.611 ± 0.003	101.83	0.49
	Second day	0.400	0.405 ± 0.002	101.25	0.49
		0.500	0.504 ± 0.005	100.80	0.99
		0.600	0.598 ± 0.001	99.67	0.17
	Third day	0.400	0.399 ± 0.004	99.75	1.00
		0.500	0.498 ± 0.002	99.60	0.40
		0.600	0.593 ± 0.002	98.83	0.34
ATV	First day	0.400	0.403 ± 0.001	100.75	0.25
		0.500	0.509 ± 0.003	101.80	0.59
		0.600	0.599 ± 0.002	99.83	0.33
	Second day	0.400	0.394 ± 0.004	98.50	1.01
		0.500	0.501 ± 0.003	100.20	0.60
		0.600	0.607 ± 0.001	101.67	0.16
	Third day	0.400	0.403 ± 0.002	100.75	0.50
		0.500	0.509 ± 0.005	101.80	0.98
		0.600	0.604 ± 0.003	100.67	0.50

a Values are the mean of three determinations (n = 6).

##### 3.2.4.2 Repeatability

Repeatability states the deviation of measurement results for the same sample under the same experimental conditions within a short period. Ten replicates were made from the sample of concentration 100% (0.50% w/w) and measured on the same day. Then, repeatability of the of this study was assessed after determining the AUC of ten replicates, and calculating the mean, standard deviation, and RSD of the ten replicates, with results shown in Table 6.

**Table 6:** Repeatability data of AML and ATV.

Replicate No.	AUC values of AML			AUC values of ATV		
	AUC of 0.4	AUCs of 0.5	AUCs of 0.6	AUC of 0.4	AUCs of 0.5	AUCs of 0.6
1	4.44	5.52	6.56	6.58	8.21	9.53
2	4.49	5.66	6.62	6.56	8.17	9.56
3	4.54	5.58	6.58	6.67	8.24	9.61
Mean	4.49	5.59	6.59	6.60	8.21	9.57
SD	0.041	0.057	0.025	0.048	0.029	0.033
RSD (%)	0.91	1.03	0.38	0.72	0.35	0.34

Note: AUC: area under the curve of the selected peak; SD: standard deviation; RSD: relative standard deviation.

From the precision test results above, it was concluded that the FTIR method is precise for both APIs since the method has met the requirements of precision. The results obtained on the same day and on three days had a statistical RSD ≤2%.

#### 3.2.5 LOD and LOQ

The limit of detection (LOD) is defined as the lowest amount of an analyte in the sample that can be detected by a method of analysis despite determining the exact quantity. Whereas, the limit of quantification (LOQ) is the smallest quantity that can be quantified with acceptable precision and accuracy. In this method, for AML obtained: LOD = 0.1263 mg.gr^−1^ (0.01263 %w/w) and LOQ = 0.421 mg.gr^−1^ (0.0421% w/w). While, for ATV, the LOD was 0.1226 mg.gr^−1^ (0.01226% w/w), and the LOQ was 0.409 mg.gr^−1^ (0.0409%w/w). These values indicate the high sensitivity of the proposed method.

Finally, it was concluded that the proposed method has met the acceptance criteria of parameters of validation. Therefore, the proposed analysis method can be used for the determination of AML and ATV simultaneously on the sample pharmaceutical tablets.

### 3.3 Application of the FTIR method in the determination of AML and ATV in pharmaceutical dosage forms

After the FTIR method has been validated, then the calibration models were applied to determine AML and ATV content in the eight commercial dosage forms of Amlostine® tablets that, were stated to contain different ratios of AML and ATV per tablet (5/10, 5/20, 5/40, 5/80, 10/10, 10/20, 10/40, and 10/80 mg). Assay results were recorded in Table 7.

**Table 7:** Results for the AML and ATV concentration found in various strengths tablets.

Sample	AML labeled mg/tab	AML Found mg/tab ± SD^a^	Mean Recovery (%)	ATV labeled mg/tab	ATV Found mg/tab ± SD^a^	Mean Recovery (%)
Sample 01	5	4.71 ± 0.076	94.20	10	9.88 ± 0.131	98.80
Sample 02	5	4.77 ± 0.107	95.40	20	20.04 ± 0.152	100.20
Sample 03	5	4.89 ± 0.111	97.80	40	39.27 ± 0.351	98.17
Sample 04	5	5.03 ± 0.200	100.60	80	79.63 ± 0.666	99.54
Sample 05	10	9.66 ± 0.106	96.60	10	10.00 ± 0.305	100.00
Sample 06	10	9.84 ± 0.170	98.40	20	19.74 ± 0.254	98.70
Sample 07	10	10.05 ± 0.436	100.50	40	40.424 ± 0.557	101.06
Sample 08	10	9.99 ± 0.087	99.90	80	80.57 ± 1.150	100.71

a Average of six determinations.

The recovery of AML by the proposed method was in the range of 94.62–100.6% while, the recovery of ATV was in the range of 98.175–101.06% of the labeled amount, so the sample of AML and ATV in combined formulation met the criteria of the USP pharmacopeia requirement. It states the range of 90–110% for AML and 94.5–105% for ATV of the levels declared in the packaging [8]. The values found for AML and ATV concentration indicate good applicability of the method for the analysis of tablet formulations. Also, values of RSD lower than 2% have proved the precision of formulation tablets analysis.

### 3.4 Statistical analysis

The results obtained from applying the FTIR method for determining AML and ATV in marketed formulations were statistically compared to the HPLC method [37] for T- and F-tests at confidence level as shown in Table 8.

**Table 8:** Statistical comparison between the results acquired by the FTIR method and HPLC method for the determination of AML and ATV in marketed formulations.

Labeled content AML/ATV (mg)		Recovery^a^ (%) ± SD		t-value^b^	F-value^c^
		Proposed method	Developed method		
5/10	AML ATV	100.31 ± 0.167 100.12 ± 0.241	99.69 ± 0.452 101.24 ± 0.322	0.114 0.521	7.326 1.784
5/20	AML ATV	100.10 ± 0.341 100.56 ± 0.311	98.57 ± 0.676 98.69 ± 0.530	1.826 1.113	3.930 2.904
10/20	AML ATV	99.83 ± 0.251 99.43 ± 0.715	100.19 ± 0.421 100.77 ± 0.721	0.134 0.511	2.813 1.017
10/40	AML ATV	100.34 ± 0.111 101.12 ± 0.122	101.02 ± 0.237 99.59 ± 0.150	0.467 0.430	4.559 1.512

a Average of five determinations; was calculated considering the stated amount of APIs reported by the manufacturer.

b Tabulated value of t at 95% confidence limit is 2.776.

c Tabulated value of F at 95% confidence limit is 19.250.

The calculated T- and F- values were less than the tabulated values which indicates that there is no significant difference between the FTIR and HPLC methods concerning accuracy and precision, which mirrors the suitability of the FTIR method for the quantification of AML and ATV in marketed formulations.

## 4 Conclusion

FTIR-AUC method has a lot of advantages for the determination of pharmaceutical samples which is inexpensive and eco-friendly, so it could be used as an alternative method for the determination of AML and ATV simultaneously in the presence of excipients, so it could be used easily for quality assessment/quality control (QA/QC) of the active ingredients like amlodipine and atorvastatin in the pharmaceutical preparations. The good applicability of the FTIR method can open the way for more studies of different drug combinations.

## Author contribution statement

Sherwan Ibesh: Performed the experiments; Wrote the paper.

Yaser Bitar: Conceived and designed the experiments.

Saleh Trefi: Analyzed and interpreted the data; Contributed reagents, materials, analysis tools or data.

## Funding statement

This research did not receive any specific grant from funding agencies in the public, commercial, or not-for-profit sectors.

## Data availability statement

Data included in article/supplementary material/referenced in article.

## Declaration of interest’s statement

The authors declare no conflict of interest.
